# Meshed Split-Thickness Autograft With a Viable Cryopreserved Placental Membrane Overlay for Lower-Extremity Recipient Sites With Increased Risk of Graft Failure

**Published:** 2018-07-05

**Authors:** Michael A. Lavor, Georgina M. Michael, Yeabsera G. Tamire, Nicole D. Dorofee

**Affiliations:** ^a^Department of Surgery; ^b^Wound and Hyperbaric Treatment Center, Tucson Medical Center, Tucson, AZ; ^c^Department of Medical Affairs, Osiris Therapeutics, Inc, Columbia, MD; ^d^University of Arizona- College of Science, Tucson, AZ

**Keywords:** split-thickness skin grafting, viable placental membrane, chronic wound, autologous tissue transfer, autograft failure

## Abstract

**Introduction:** Meshed split-thickness skin grafting represents a rapid and effective technique for surgical wound closure. Factors such as ongoing inflammation, microbial colonization, and a poorly vascularized wound bed increase the rate of skin autograft failure up to 33%. Because of the inherent angiogenic, anti-inflammatory, antimicrobial, and antifibrotic properties of human placental membranes, the complementary use of human placental membranes may promote graft survival and improve success rate for complete ulcer resolution. **Methods:** In this case series, a viable cryopreserved placental membrane was used as a meshed split-thickness skin grafting overlay in 6 high-risk patients with various comorbidities and recalcitrant nonhealing lower-extremity wounds. **Results:** The mean size of grafted wounds was 130.3 cm^2^. The average graft take-rate by postoperative days 10 to 14 was 92.5%, with complete epithelialization of all skin graft interstices observed between days 10 and 21. Transplanted autograft tissues did not lyse or dissolve, and sites remained free of infection and maceration throughout postoperative follow-up. Complete wound closures remained intact at the 12-month follow-up visit. **Discussion:** Thus far, our clinical experience has warranted the complementary use of viable cryopreserved placental membrane and meshed split-thickness skin grafting to reduce the need for repeat surgical interventions or prolonged local wound care due to graft loss or failure in high-risk patients.

In the United States alone, chronic wounds affect 6.5 million patients and contribute more than $50 billion a year to rising health care expenditures.^1^^,^^2^ Despite multidisciplinary efforts that include advanced therapies, one-third of chronic wounds remain refractory and will require surgical intervention.^3^^,^^4^ Meshed split-thickness skin grafting (mSTSG) currently represents the most rapid and effective technique for surgical wound closure when established clinical pathways fail to overcome host factors that impact healing.^5^ Microbial imbalance, inflammation, excessive moisture or exudate, and suboptimal perfusion in chronic wound beds represent risk factors for autologous tissue transfer loss.^6^^,^^7^

Ongoing microenvironmental abnormalities in chronic wounds may result in partial mSTSG failure with typically less than 50% residual graft present at the 3-month follow-up.^8^ Despite the general success rate associated with the mSTSG procedure, a significant portion of patients with recalcitrant lower-extremity wounds and chronic comorbid conditions—diabetes, peripheral arterial disease, obesity, venous insufficiency, advanced age—will experience partial or complete postoperative mSTSG failure.^8^^,^^9^ Although incorporation of negative-pressure wound therapy (NPWT) improves the likelihood of initial mSTSG survival by reducing infection, shear stress, and edema, rates of complete postoperative autograft failure may still be as high as 33%.^10^

Since the early 20th century, human placental membranes (hPMs) have been clinically integrated as a biological dressing for a broad variety of acute and chronic wounds.^11^^,^^12^ A 3-dimensional (3D) collagen-rich extracellular matrix, growth factors, and endogenous neonatal fibroblasts, epithelial cells, and mesenchymal stem cells represent native membrane components.^13^ Because of their ability to downregulate inflammation and prevent tissue fibrosis, hPMs have been successfully adopted into various soft tissue reconstructive procedures where minimizing adhesions and reducing scar formation are critical for successful patient outcomes.^14^ hPMs preserved in their native-most state also exhibit a variety of properties—anti-inflammatory, antioxidant, and angiogenic—that play an important role in coordinating tissue repair.^15^ In particular, the antimicrobial and epithelialization-promoting characteristics found in hPMs make them an attractive surgical adjunct to the mSTSG procedure.^11^^,^^16^

In this case series, we explore the use of a viable cryopreserved placental membrane (vCPM; Grafix PRIME; Osiris Therapeutics, Inc, Columbia, MD) allograft in the management of lower-extremity wounds at high risk for autograft recipient failure. The authors describe the use of vCPM as an overlay in conjunction with standard surgical protocols for mSTSG and report clinical outcomes related to autograft survival and complete ulcer resolution at long-term follow-up.

## MATERIALS AND METHODS

### Viable cryopreserved placental membrane

This aseptically processed vCPM is a commercially available product used in the management of acute and chronic wounds. vCPM is fully tested, stored, and distributed according to the US Food and Drug Administration and the American Association of Tissue Banks requirements and has a 3-year shelf life at −80°C.^17^ This point-of-care placental allograft retains a 3D extracellular matrix, resident growth factors, viable cells—epithelial cells, fibroblasts, mesenchymal stem cells—which are inherent properties of fresh placental tissue.^17^ vCPM's inner pouch is sterile, allowing its use in the operating room.^17^ Once thawed according to the product insert, the tissue can be held in a sterile rinse basin with normal sterile saline 0.9% for up to 1 hour.^17^ Tissues may be placed within operative sites as a cover or wrap and optionally secured in place using sutures.

### Study design and patients

The purpose of this investigator-initiated study was to report the clinical outcomes of a 1-year evaluation for vCPM as an adjunct to standard surgical treatment protocols. Institutional approval was granted for the retrospective collection and presentation of material and data. De-identified data sets, consistent with the Health Insurance Portability and Accountability Act of 1996 (HIPAA), were collected from electronic health records. All procedures followed were in accordance with the ethical standards of the responsible committee on human experimentation (institutional and national) and with the Helsinki Declaration of 1975, as revised in 2008.

In this study, patients (n = 6) with recipient sites considered to be at high risk for autograft failure were identified and managed using the vCPM-mSTSG technique. Informed written consent was obtained from all individual participants included in this study. Recipient sites were characterized to be at high risk based on the following criteria: (1) presence of complex comorbid disease states known to impact lower-extremity wound healing, that is, diabetes mellitus, peripheral arterial disease, venous insufficiency, congestive heart failure with lower-extremity edema, obesity, or autoimmune disorder; in addition to, (2) long-term (>1 year) failure to demonstrate measureable reductions in wound size using good wound care (GWC) practices; and (3) failure to achieve delayed primary closure through surgical interventions, that is, previous failure of autologous tissue transfers. For the purposes of this evaluation, a postoperative complication was defined as total autograft failure or surgical site infection.

### Preoperative evaluation and management

Preoperative patient and wound assessment followed standard clinical practice guidelines to medically manage comorbidities and address underlying issues such as infection, pressure, edema, ischemia, and exudate. After a minimum of 2 weeks of GWC that included regular sharp debridement and local cleansing, wound swabs were taken for evaluation of wound pathogens prior to surgical intervention. Qualitative or semiquantitative swab cultures with Gram stain were considered sufficient to identify and manage pathogens of primary concern (methicillin-resistant *Staphylococcus aureus* [MRSA], *Pseudomonas aeruginosa*, *Proteus mirabilis*). If warranted, a prescribed course of topical antimicrobials or systemic antibiotics was administered preoperatively.

### Surgical technique with vCPM overlay

**Figure 1:**
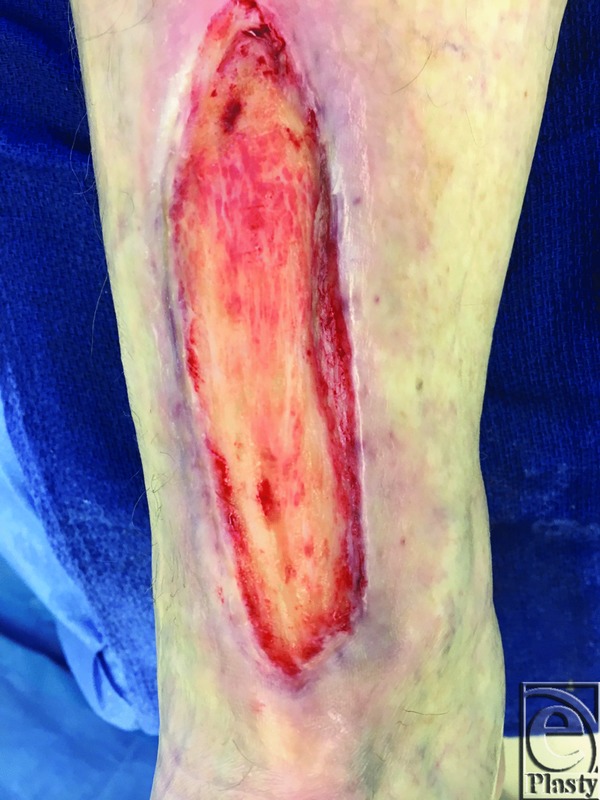
Patient 2: Chronic anterior ankle wound (48 cm^2^) prior to excisional debridement with mSTSG and vCPM overlay.

Following induction of patient anesthesia, standard surgical protocols for mSTSG were followed. Once the recipient site had been carefully debrided using Versajet II Hydrosurgery System (Smith & Nephew, London, UK) and selective use of a weck blade, the area was recleansed, redraped, and readied for the autografting portion of the procedure (Fig 1). At this time, vCPM was prepared according to the package insert and kept on the surgical table in a sterile bath of normal saline solution (NSS 0.9%). Next, a thin STSG graft of 0.12-mm thickness was harvested from either the donor calf or thigh region using an electric dermatome (Zimmer Biomet, Warsaw, IN). The harvested tissue was meshed on the Dermacarrier (Zimmer Biomet) in a 1:1.5 ratio.

**Figure 2:**
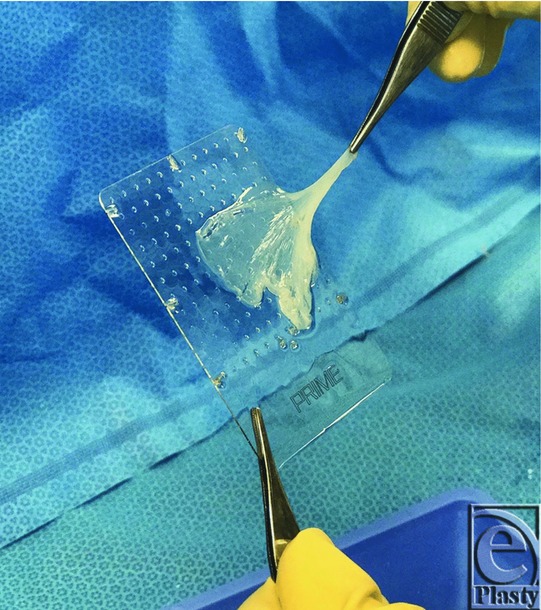
Removal of the first vCPM from the applicator card (25 cm^2^) postthaw, using sterile technique.

**Figure 3:**
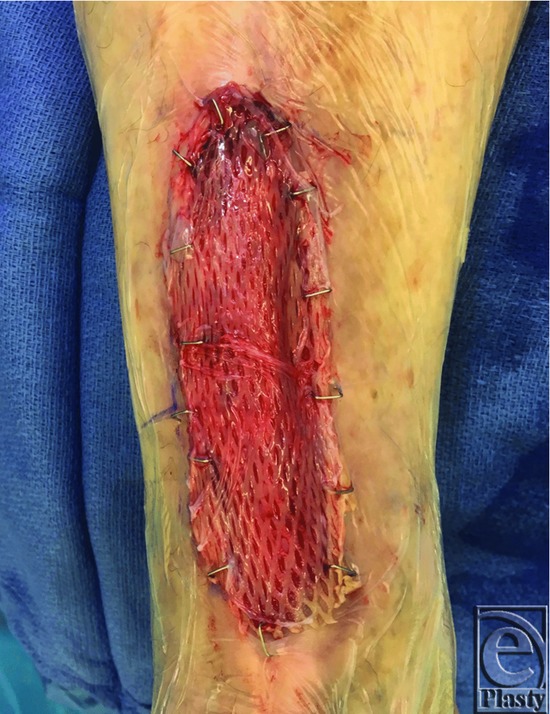
The vCPM allograft was easily applied as a cover over the irregular surface of the mSTSG; additional staples were not required.

**Figure 4:**
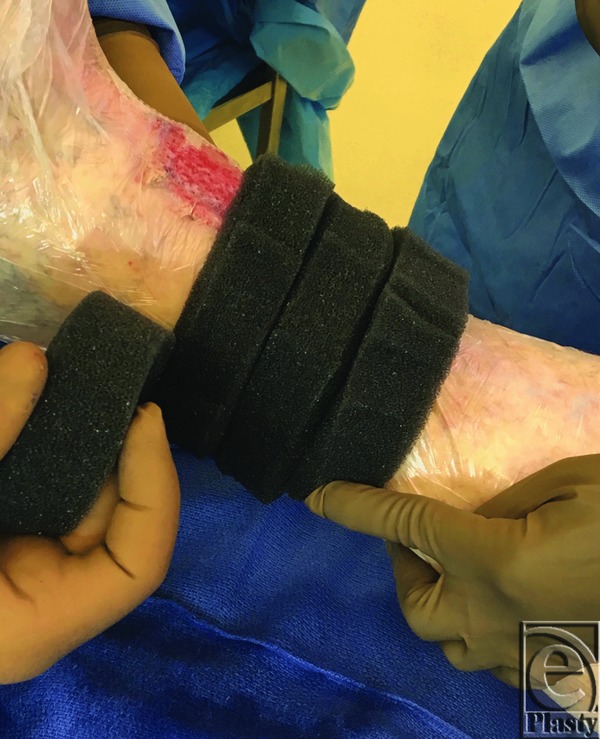
Negative-pressure wound therapy was applied to the recipient site using black foam dressing placed circumferentially in a spiral manner.

Once the mSTSG had been secured to the recipient wound bed using skin staples, the vCPM was carefully detached from the plastic applicator (Fig 2) and applied as a cover atop the entire autografted area (Fig 3). To protect the vCPM and prevent NPWT foam adhesion to the placental allograft tissue, a primary nonadherent dressing (Mepitel One; Mölnlycke Health Care, Norcross, Ga) was placed on the top of the vCPM to keep the placental tissue in place as an mSTSG overlay. Additional vCPM fixation was not required. Finally, NPWT (V.A.C.; Acelity, San Antonio, TX) was applied to the recipient site using black GranuFoam dressing placed circumferentially in a spiral manner (Fig 4). Suction was set to a continuous cycle of −75 mm Hg, as prescribed by clinical guidelines for target pressure application to meshed graft sites.^18^ Pressure recommendations remain at −75 mm Hg to reduce shear forces, keep the mSTSG in place, and act as a compression dressing to decrease immediate postoperative edema. Patients were discharged home with instructions to keep the extremity elevated, to ambulate using only limited partial weight-bearing, and to leave the NPWT dressings undisturbed until the first postoperative visit.

### Postoperative graft assessment and care

NPWT was discontinued at the first postoperative visit on days 7 to 10. Primary nonadherent dressings and surgical staples were also removed for surgical site assessment. During the second postoperative visit on days 10 to 14, the take-rate of mSTSG was quantified in percentages based on the total surface area of wound (cm^2^). Areas with good mSTSG adherence to the recipient wound bed with or without interstitial granulation were considered viable. The areas of necrotic, nonadhered, and lysed portions of grafted skin were considered as graft-loss. These areas were measured, and the percentage of graft loss was calculated relative to the total surface area of the wound.

Next, the wounds were cleansed with 4% chlorhexidine gluconate. The primary nonadherent dressings were replaced and covered with a layer of NSS 0.9% moistened gauze. Starting proximal to the metatarsal heads of the foot, the entire lower leg was wrapped in 4.5-inch bandage rolls (Kerlix; Medtronic, Minneapolis, MN), followed by a lightly compressive 3-inch self-adherent wrap (3M Coban, St Paul, MN). Patients were instructed to continue elevation of the affected limb and to limit fully weight-bearing ambulation. This cleansing and dressing change protocol was repeated on a weekly basis until complete epithelialization of meshed interstices and graft maturation was achieved.

## RESULTS

**Figure 5:**
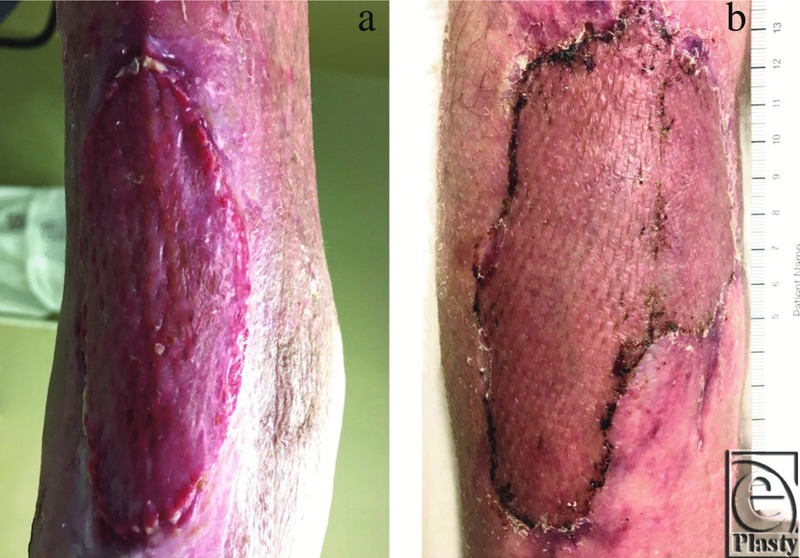
Postoperative examples of high-risk sites grafted with mSTSG-vCPM. (a) Patient 1: 42-cm^2^ chronic posterior ankle wound with exposure of Achilles tendon, postoperative day 21. (b) Patient 3: 100-cm^2^ chronic anterolateral ulcer with exposed subcutaneous fat, postoperative day 14.

Patient demographics and clinical outcomes using the mSTSG-vCPM overlay technique are summarized in Table 1. The average age of study subjects (n = 6) was 73.2 years (4 males, 2 females). The mean size of grafted wounds was 130.3 cm^2^. The average graft take-rate by postoperative days 10 to 14 was 92.5%, with complete epithelialization of all skin graft interstices observed between days 10 and 21 (Figs 5 *a* and 5*b*). Wounds were free of infection, edema, or maceration throughout postoperative follow-up. All successfully grafted sites matured, and wounds remained closed at the 3-, 6-, 9-, and 12-month follow-up visits. There were no postoperative complications attributed to the use of vCPM in conjunction with standard surgical protocols for mSTSG noted amongst study subjects.

**Figure 6:**
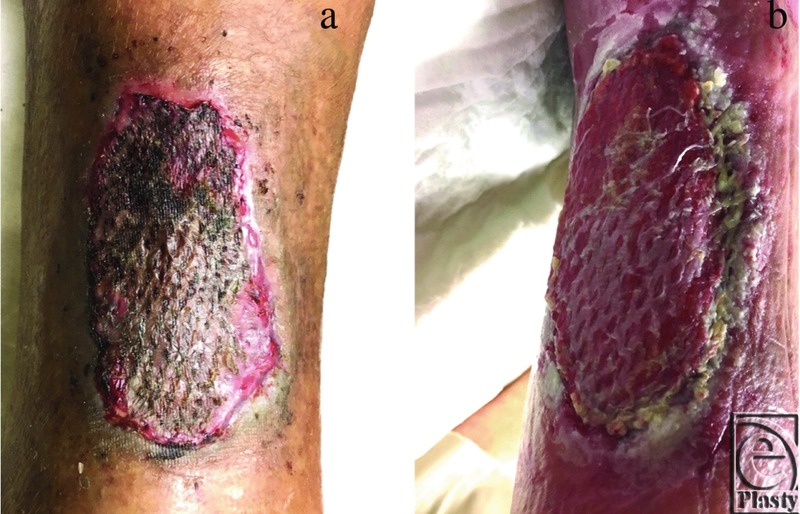
Examples of previous mSTSG failures in high-risk cases grafted without vCPM overlay. (a) Drying, necrosis, and lysing of transferred autograft tissues with retracted and dissolved edges. (b) Periwound maceration with dissolving and lysing of the transferred autograft tissues.

Prior to the adjunct of vCPM, all wounds—including those considered to be at high risk—were managed with the perioperative protocol using mSTSG alone in conjunction with 7 to 14 days of NPWT. Even with the initial NPWT, transferred skin was notably necrotic with poor adherence, contracted edges, or lysed portions, which led to partial or complete sloughing off of the graft and near total mSTSG failure during the immediate postoperative time frame (Figs 6 *a* and 5*b*). These wounds required either continued periods of prolonged local GWC or repeat surgical autografting procedures—a challenging pursuit in patients with limited and poor tissue quality at remaining donor sites.

### Case example

**Figure 7:**
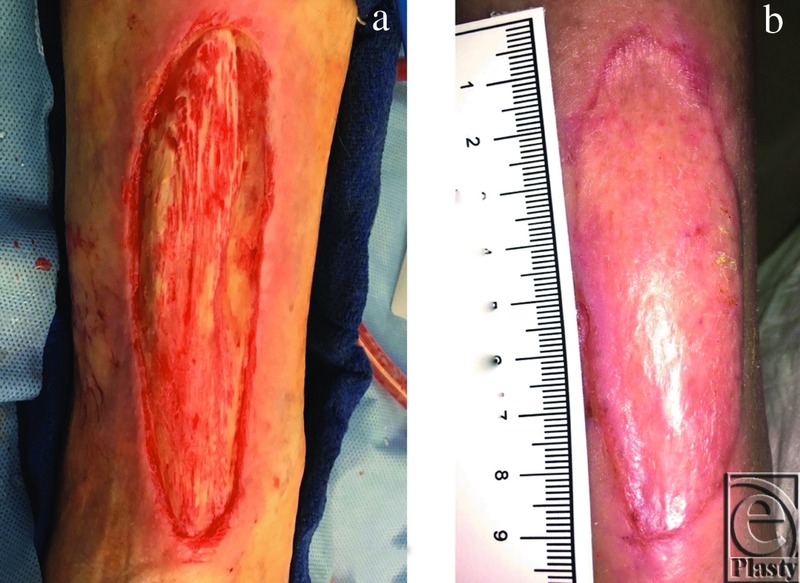
Case example. Patient 6. (a) Chronic nonhealing posterior ankle wound with necrosis of muscle, following excisional debridement in preparation for mSTSG with vCPM overlay. (b) Follow-up visit at 3-months postoperative mSTSG technique.

A 79-year old man (patient 6, Table 1) presented with a chronic nonhealing ulcer of the left posterior leg and ankle with necrosis of his muscle. The wound started with a blister caused by an offloading boot. The open area subsequently became infected after repeated traumas. Medical history was significant for chronic venous insufficiency, varicose veins with ulcer and inflammation, coronary artery disease, chronic obstructive pulmonary disease, hypertension, daily oral steroid medication, MRSA infection, and poor healing. After experiencing 3 previously failed surgical interventions (without the use of vCPM), the patient was scheduled for an mSTSG with vCPM overlay. The wound measured 44 cm^2^ following excisional debridement of the subcutaneous tissue and muscle (Fig 7*a*). Following fixation of the mSTSG, vCPM, NPWT, and a posterior splint, the patient was discharged home and evaluated at the prescribed postoperative intervals. At the 3-month follow-up visit, wound closure remained intact (Fig 7*b*).

## DISCUSSION

The use of fresh amnion as a protective meshed-skin autograft dressing has been previously described. In 1984, Lin et al^19^ observed increased rates of complete burn healing (days 6-10) and reduced pain and edema in the fresh amnion-mSTSG grafted group versus the control. Patients receiving mSTSG alone experienced reduced graft survival and increased hemorrhage, mechanical disruption, and pain. In 1995, Subrahmanyam^20^ used fresh amnion to cover microskin grafts to expand the area covered with a limited amount of harvested STSG tissue. A 15-fold expansion of amnion-covered microskin grafts was achieved in all cases, and 75.0% of lower-extremity wounds completely healed by day 9.^20^ More recently, Mohammadi and colleagues^21^^,^^22^ compared the rate of and time to complete graft take in fresh amnion-covered mSTSG sites versus control wounds treated with mSTSG alone. In 2 separate studies, outcomes for wounds treated with the fresh amnion-mSTSG combination demonstrated statistically significant improvement over control (*P* < .001).^21^^,^^22^ Until now, there has been limited reporting on the use of commercially processed hPMs used in conjunction with mSTSG.

Our 1-year evaluation period of the vCPM-mSTSG technique demonstrated positive outcomes. During postoperative evaluation, mSTSG tissues covered with a vCPM overlay did not lyse or dissolve and sites remained free of infection and maceration. Autologous graft loss due to infection is one of the primary causes for postoperative “melting” and destruction of the tissues transplanted into chronic wounds, even when the positive cultures occurred up to 12 weeks before surgery.^23^ According to the literature, the presence of *Pseudomonas aeruginosa* or *Staphylococcus aureus* serves as a predictive factor for skin graft failure and is associated with increasing reoperation rates by 4.2 times.^23^^,^^24^ In vitro studies have demonstrated that aseptically cryopreserved hPMs retain the intrinsic antimicrobial activities of fresh hPMs, showing statistically significant log reductions in both *Pseudomonas aeruginosa* and *Staphylococcus aureus* (*P* < .001).^25^

In our study, high-risk patients experienced improved graft take-rates at postoperative days 7 to 10, complete epithelialization of graft interstices by day 21, and no wound reoccurrence during the first year of follow-up. Although vCPM may increase supply-related expenditures for the surgery, the authors considered the cost justifiable in patients at a high risk for skin graft loss, as repeat surgical interventions were not required to achieve definitive closure in this group. Our initial clinical experience has warranted the ongoing use of vCPM in the high-risk patient population, as it reduces long-term costs associated with repeated surgical interventions or prolonged local wound care secondary to skin graft loss or failure. The retrospective nature, lack of control arm, small sample size, and short-term presence of NPWT—a variable known to aid with graft take—are limitations of the study. The authors take caution while interpreting these results, and it is recognized that further studies are required to support the initial findings.

## Figures and Tables

**Table 1:** Clinical outcome of mSTSG with vCPM overlay*

Patient	Age (sex)	Comorbidities	Wound type	Grafted site, cm^2^	Graft take (%) at second postoperative visit^†^	Closure intact at 1-y follow-up
**1**	89 (M)	Diabetes Peripheral arterial disease Coronary artery disease Pulmonary fibrosis Daily high-dose steroid medication Neuropathy	Chronic ulcer of posterior ankle with Achilles tendon exposure secondary to pressure	42.0	100.0	Yes
**2**	67 (M)	Diabetes Obesity Hypertension DVT Hodgkin lymphoma Neuropathy	Chronic anterior ankle ulcer with tibialis tendon exposure secondary to trauma	48.0	85.0	Yes
**3**	81 (F)	Raynaud disease Chronic venous insufficiency COPD Hypercholesterolemia Hypertension	Chronic anterolateral leg ulcer with exposure of fat layer secondary to trauma	122.0	100.0	Yes
**4**	48 (M)	Diabetes Necrotizing fasciitis Peripheral arterial disease Severe calcification of LEA Neuropathy Cancer	Chronic necrotic plantar foot ulcer with exposed muscle, fascia, tendon secondary to necrotizing fasciitis infection	449.0	100.0	Yes
**5**	75 (F)	Cardiomyopathy Mitral valve replacement Congestive heart failure DVT Breast cancer	Chronic necrotic wound of lateral ankle secondary to surgical dehiscence of joint fusion	77.0	90.0	Yes
**6**	79 (M)	Chronic venous insufficiency Varicose veins with ulcer and inflammation Coronary artery disease COPD Hypertension Daily steroid medications	Chronic wound posterior ankle with necrosis of muscle secondary to trauma	44.0	80.0	Yes

*M indicates male; DVT, deep vein thrombosis; F, female; COPD, chronic obstructive pulmonary disease; and LEA, lower-extremity arteries.

†Second postoperative visit occurred on days 10 to 14.
